# Oral rivaroxaban for Japanese patients with symptomatic venous thromboembolism – the J-EINSTEIN DVT and PE program

**DOI:** 10.1186/s12959-015-0035-3

**Published:** 2015-01-17

**Authors:** Norikazu Yamada, Atsushi Hirayama, Hideaki Maeda, Satoru Sakagami, Hiroo Shikata, Martin H Prins, Anthonie WA Lensing, Masaharu Kato, Junichi Onuma, Yuki Miyamoto, Kazuma Iekushi, Mariko Kajikawa

**Affiliations:** Department of Cardiology and Nephrology, Mie University Graduate School of Medicine, Mie, 514-8507 Japan; Division of Cardiology, Department of Medicine, Nihon University School of Medicine, Tokyo, Japan; Division of Cardiovascular, Respiratory and general surgery, Nihon University School of Medicine, Tokyo, Japan; Department of Cardiology, National Hospital Organization, Kanazawa Medical Center, Kanazawa, Japan; Department of Cardiovascular Surgery, Kanazawa Medical University, Ishikawa, Japan; Maastricht University Medical Center, Maastricht, The Netherlands; Bayer HealthCare Pharmaceuticals, Wuppertal, Germany; Bayer Yakuhin Ltd, Osaka, Japan

**Keywords:** Deep vein thrombosis, Japan, Pulmonary embolism, Randomized trial, Rivaroxaban, Unfractionated heparin, Venous thromboembolism, Warfarin

## Abstract

**Background:**

The global EINSTEIN DVT and PE studies compared rivaroxaban (15 mg twice daily for 3 weeks followed by 20 mg once daily) with enoxaparin/vitamin K antagonist therapy and demonstrated non-inferiority for efficacy and superiority for major bleeding. Owing to differences in targeted anticoagulant intensities in Japan, Japanese patients were not enrolled into the global studies. Instead, a separate study of deep vein thrombosis (DVT) and/or pulmonary embolism (PE) in Japanese patients was conducted, which compared the Japanese standard of care with a reduced dose of rivaroxaban.

**Methods:**

We conducted an open-label, randomized trial that compared 3, 6, or 12 months of oral rivaroxaban alone (10 mg twice daily or 15 mg twice daily for 3 weeks followed by 15 mg once daily) with activated partial thromboplastin time-adjusted intravenous unfractionated heparin (UFH) followed by warfarin (target international normalized ratio 2.0; range 1.5–2.5) in patients with acute, objectively confirmed symptomatic DVT and/or PE. Patients were assessed for the occurrence of symptomatic recurrent venous thromboembolic events or asymptomatic deterioration and bleeding.

**Results:**

Eighty-one patients were assigned to rivaroxaban and 19 patients to UFH/warfarin. Three patients were excluded because of serious non-compliance issues. The composite of symptomatic venous thromboembolic events or asymptomatic deterioration occurred in 1 (1.4%) rivaroxaban patient and in 1 (5.3%) UFH/warfarin patient (absolute risk difference, 3.9% [95% confidence interval, -3.4–23.8]). No major bleeding occurred during study treatment. Clinically relevant non-major bleeding occurred in 6 (7.8%) patients in the rivaroxaban group and 1 (5.3%) patient in the UFH/warfarin group.

**Conclusions:**

The findings of this study in Japanese patients with acute DVT and/or PE suggest a similar efficacy and safety profile with rivaroxaban and control treatment, consistent with that of the worldwide EINSTEIN DVT and PE program.

**Trial registration:**

Clinicaltrials.gov: NCT01516840 and NCT01516814.

**Electronic supplementary material:**

The online version of this article (doi:10.1186/s12959-015-0035-3) contains supplementary material, which is available to authorized users.

## Background

Acute deep vein thrombosis (DVT) and pulmonary embolism (PE) are common disorders in the Western world [1-3], and treatment recommendations are based on the results of large randomized trials [4]. In Japan, the incidence rates of DVT and PE have not been well established and the clinical management of acute venous thromboembolism (VTE) is not well defined. The Japan VTE Treatment Registry (JAVA) evaluated more than 1000 consecutive patients with an objectively confirmed, symptomatic acute PE, symptomatic acute DVT, or asymptomatic acute proximal DVT [5]. VTE management was characterized by a highly aggressive strategy in the acute phase, with frequent use of inferior vena cava filter insertion (40.6%) and thrombolysis (21.1%), whereas anticoagulation was characterized by targeting sub-therapeutic levels of unfractionated heparin (UFH) and warfarin.

The recommended regimen of anticoagulant therapy for VTE in Japanese guidelines differs from that in other parts of the world, consisting of UFH infusion (instead of subcutaneous low molecular weight heparin [LMWH]) overlapping with and followed by warfarin, with a target international normalized ratio (INR) range of 1.5–2.5 instead of 2.0–3.0 [6]. This regimen is complex, owing to the need for intravenous administration of UFH and laboratory monitoring and dose adjustment for both UFH and warfarin [7,8].

Rivaroxaban is an orally active, specific, direct inhibitor of activated Factor Xa that has predictable pharmacokinetics and pharmacodynamics [9]. It has a half-life of 5–9 hours in healthy young subjects and 11–13 hours in elderly subjects, in addition to a dual mode of elimination: two-thirds of the drug is metabolized by the liver, with one-third excreted as unchanged drug by the kidneys. Rivaroxaban was evaluated in large, global clinical trials in patients with symptomatic DVT and/or PE using a fixed-dose oral regimen of 15 mg twice daily for the initial 3 weeks followed by 20 mg once daily thereafter [10,11]. Rivaroxaban was non-inferior to standard treatment and was associated with a reduced incidence of major bleeding events. In addition, the Kaplan–Meier curves for recurrence with this regimen and with standard therapy were almost identical, with fewer major bleeding events in the rivaroxaban group during the initial treatment phase [12,13]. Owing to the different anticoagulant regimens used in Japan, Japanese patients were not enrolled into the global EINSTEIN DVT and PE trials.

We conducted the Japanese (J)-EINSTEIN DVT and PE program to compare rivaroxaban with the Japanese standard of care; however, in line with Japanese clinical practice, we used a lower dose of rivaroxaban than that used in the global EINSTEIN DVT and PE studies.

## Methods

### Study design and patients

The J-EINSTEIN DVT and PE program had an open-label, randomized design and compared 3, 6, or 12 months of rivaroxaban with UFH and warfarin in patients older than 20 years who had acute, objectively confirmed symptomatic proximal DVT and/or PE. Patients were excluded if they had received heparin or fondaparinux treatment for longer than 48 hours or more than a single dose of warfarin. Other exclusion criteria were: thrombectomy, insertion of a caval filter, or use of a fibrinolytic agent for the current episode; any contraindication listed in the local labeling of UFH or warfarin or another indication for the use of UFH or warfarin; a creatinine clearance <30 ml/min; significant hepatic disease or alanine aminotransferase >3 times the upper limit of normal; bacterial endocarditis; active bleeding or a high risk of bleeding contraindicating treatment with UFH or warfarin; a systolic blood pressure of more than 180 mm Hg or a diastolic blood pressure of more than 110 mm Hg; childbearing potential without proper contraceptive measures, pregnancy, or breast-feeding; concomitant use of strong cytochrome P450 3A4 inhibitors (i.e. azole-antimycotics or HIV protease inhibitors); and a life expectancy of less than 3 months.

The program was sponsored by Bayer Yakuhin Ltd, Japan. The protocols were approved by the local institutional review boards, and written informed consent was obtained from all patients. The data were collected and maintained by Bayer Yakuhin. An independent data and safety monitoring board periodically reviewed outcomes. A central independent committee whose members were unaware of treatment allocation assessed all baseline and repeat imaging tests and all suspected efficacy and bleeding events.

### Treatment regimens and randomization

DVT-only patients received rivaroxaban 10 mg or 15 mg twice daily for a total of 3 weeks in a double-blind fashion, followed by open-label rivaroxaban 15 mg once daily. All PE patients (with or without DVT) received rivaroxaban 15 mg twice daily for 3 weeks followed by 15 mg once daily. Patients assigned to control treatment received intravenous UFH, with the dose adjusted to prolong the activated partial thromboplastin time to 1.5–2.5-fold that of controls, for at least 5 days, overlapping with and followed by INR (range 1.5–2.5)-titrated warfarin. UFH was discontinued when the INR was 1.5 or more for two consecutive measurements at least 24 hours apart. Initially, the INR was measured every 2–3 days and, when stable, at least once per month. Treatment was continued for 3, 6, or 12 months, as decided by the treating physician. The use of non-steroidal anti-inflammatory drugs and antiplatelet agents was discouraged [14]; however, both acetylsalicylic acid (up to 100 mg per day) and clopidogrel (up to 75 mg per day) were allowed.

Randomization was done centrally, using an interactive web response system, with a rivaroxaban to control ratio of 4:1. Randomization was stratified for DVT or PE and the intended treatment duration. In addition, rivaroxaban patients with DVT only were further allocated in a 1:1 ratio to the two different rivaroxaban regimens for the first 3 weeks.

### Surveillance and follow-up

Patients were followed-up for the intended treatment duration and seen at fixed intervals that were identical between treatment groups. At each visit, a checklist was used to elicit information on signs and symptoms of recurrent VTE, bleeding, and adverse events. Patients were instructed to report to the study center immediately if any of these events occurred. In case of clinically suspected recurrent VTE, objective testing was required. Venous compression ultrasound and spiral computed tomography (CT) were repeated at day 22 and at the end of the 3-, 6-, or 12-month intended treatment period.

### Study outcomes

Patients were assessed for the occurrence of symptomatic recurrent VTE or asymptomatic deterioration. Symptomatic VTE was defined as symptomatic DVT or symptomatic fatal or non-fatal PE, as previously described [12]. PE was considered as the cause of death if there was objective documentation, or if death could not be attributed to a documented cause and PE could not be ruled out.

Venous ultrasound and spiral CT performed at day 22 and at the end of intended treatment were compared with baseline for changes. To allow for a quantitative assessment of the venous ultrasound, the residual diameters of the common femoral, superficial femoral, and popliteal veins were measured under full compression. Diameters obtained at day 22 and at the end of intended treatment were compared with baseline and scored as normalized, improved (i.e. a decrease of 4 mm or more), unchanged, or deteriorated (i.e. an increase of 4 mm or more) [15-17]. To allow for a quantitative assessment of CT and perfusion lung scans, the percentage of vascular obstruction was calculated as described earlier [18-22]. For each scan, an estimate was made of the remaining perfusion of each of the six lobes, which was expressed as 0 (no perfusion), 0.25, 0.50, 0.75, and 1.0 (normal perfusion). The total perfusion score was the sum of the remaining perfusion of all lobes, corrected with a factor of 0.45 for the left lung and 0.55 for the right lung to reflect the difference in lung size. The lobe scores of the repeat scans were scored as normalized, improved (i.e. the perfusion defect has decreased by more than 50% compared to the baseline scan), unchanged, or deteriorated (i.e. the lobe score decreased by more than 0.25 for any individual lobe. The combined repeat ultrasound and spiral CT result at day 22 and at the end of intended treatment was considered as normalized (no thrombus in legs and lungs), improved (improved results for both legs and lungs, or improved for either legs or lungs without deterioration), unchanged (unchanged results for both legs and lungs), deteriorated (any deterioration in either legs or lungs), or not evaluable.

Bleeding was defined as major if it was clinically overt and associated with a decrease in hemoglobin levels of 2.0 g per deciliter or more or a transfusion of 2 or more units of red blood cells; or if bleeding was intracranial or retroperitoneal in nature, occurred in another critical site, or contributed to death. Clinically relevant non-major bleeding was defined as overt bleeding that did not meet the criteria for major bleeding but was associated with medical intervention, unscheduled contact with a physician, interruption or discontinuation of a study drug, or discomfort or impairment of activities of daily life [12].

### Statistical analysis

All analyses were performed on the intention-to-treat population, with the exception of bleeding outcomes, which were evaluated for the period that patients were on study medication, plus 2 days. Crude percentages and absolute differences and their 95% confidence intervals were calculated. Confidence intervals for proportions and for difference in proportions were calculated using exact methods. The mean percentage of time during which the INR was in the therapeutic range was also calculated.

## Results

### Patients

**Figure 1:**
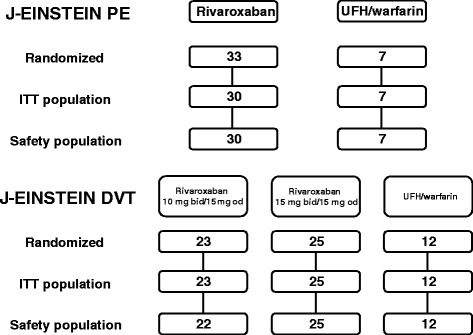
**Patient flow diagram.** bid, twice daily; ITT, intention-to-treat; od, once daily; UFH, unfractionated heparin.

Between February 2012 and December 2013, a total of 100 patients with DVT and/or PE were randomized at 39 sites; 81 patients were assigned to receive rivaroxaban and 19 were assigned to receive control therapy (Figure 1). All 3 patients from a single site were excluded from all analyses because of serious non-compliance with the protocol/Good Clinical Practice guidelines. The demographic characteristics, risk factor profile, and intended treatment duration of the patients in the treatment groups are shown in Table 1.

**Table 1:** **Baseline characteristics**

	**Rivaroxaban 10 mg bid/ 15 mg od N = 23**	**Rivaroxaban 15 mg bid/ 15 mg od N = 55**	**UFH/warfarin N = 19**
Sex, male, n (%)	16 (69.6)	25 (45.5)	10 (52.6)
Age, years, mean (SD)	65.0 (9.9)	68.8 (12.2)	63.4 (18.3)
Body weight <70 kg, n (%)	15 (65.2)	41 (74.5)	15 (78.9)
Creatinine clearance <80 ml/min, n (%)	9 (39.1)	36 (65.5)	11 (57.9)
DVT only, n (%)	23 (100.0)	25 (45.5)	12 (63.2)
Unprovoked VTE, n (%)	11 (47.8)	31 (56.4)	8 (42.1)
Provoked VTE, n (%)	12 (52.2)	24 (43.6)	11 (57.9)
Previous VTE, n (%)	0	8 (14.5)	1 (5.3)
Recent surgery or trauma, n (%)	6 (26.1)	11 (20.0)	2 (10.5)
Active cancer, n (%)	2 (8.7)	3 (5.5)	2 (10.5)
Thrombophilia, n (%)	2 (8.7)	1 (1.8)	3 (15.8)
Protein S deficiency	0	0	1 (5.3 %)
Protein C deficiency	1 (4.3)	1 (1.8)	2 (10.5)
Antithrombin deficiency	1 (4.3)	0	0
Prolonged immobilization, n (%)	3 (13.0)	8 (14.5)	4 (21.1)
Intended treatment duration			
3 months, n (%)	4 (17.4)	10 (18.2)	4 (21.1)
6 months, n (%)	12 (52.2)	26 (47.3)	9 (47.4)
12 months, n (%)	7 (30.4)	19 (34.5)	6 (31.6)
Mean treatment duration, days (SD)	191.8 (106.9)	196.6 (121.7)	199.8 (101.9)
Adherence to study treatment			
Missing n (%)	1 (4.3)	0	0
≥50% to <80%	0	0	4 (21.1)
≥80%	22 (95.7)	55 (100.0)	15 (78.9)

bid, twice daily; DVT, deep vein thrombosis; od, once daily; SD, standard deviation; UFH, unfractionated heparin; VTE, venous thromboembolism.

### Treatment and follow-up

Among rivaroxaban patients, the mean treatment duration was 195 days. In all but 1 rivaroxaban patient, tablet count revealed study medication intake above 80% (Table 1). In the control group, the mean duration of UFH treatment was 7.8 (±3.0) days, and the mean treatment duration was 200 days. The mean percentage of time during which the INR was in the therapeutic range (1.5–2.5) was 80.3%; the corresponding percentages for INRs >2.5 and <1.5 were 10.4% and 9.3%, respectively. None of the patients were lost to follow-up.

### Clinical outcomes

A single patient in the rivaroxaban group (1/78; 1.4%) developed symptomatic recurrent VTE compared with none of the 19 patients allocated to control treatment. Asymptomatic deterioration on repeat imaging at the end of intended treatment did not occur in patients assigned to rivaroxaban and was found in 1 patient (1/19; 5.3%) in the control group. As a consequence, the composite of symptomatic recurrent VTE and asymptomatic deterioration at the end of intended treatment occurred in 1 (1.4%) rivaroxaban patient and in 1 (5.3%) control patient, with an absolute risk difference of 3.9% (95% confidence interval −3.4 to 23.8).

None of the 77 patients who received rivaroxaban and none of the 19 patients who received control treatment experienced a major bleeding event while receiving study medication, whereas clinically relevant non-major bleeding occurred in 6 (7.8%) patients and 1 (5.3%) patient, respectively. Trivial bleeding events occurred in 24 (31.2%) and 7 (36.8%) of the rivaroxaban and control recipients, respectively.

In total, 3 (3.9%) of the 78 patients assigned to rivaroxaban died during the study period because of cardiac failure (n = 2) and gastrointestinal bleeding (n = 1). This fatal bleeding event occurred 3 days after discontinuation of rivaroxaban while the patient was receiving intravenous UFH and warfarin. None of the 19 patients allocated to control treatment died during the intended treatment period.

### Repeat imaging

At day 22, the combined venous ultrasound and lung imaging result in patients with DVT and/or PE showed normalization in 20 (26.7%) of the 75 rivaroxaban recipients and in 3 (15.8%) of the 19 control patients. At the end of the intended treatment period, these numbers were 44 (62.0%) of the 71 rivaroxaban recipients and 6 (31.6%) of the 19 control patients. Improvement or normalization occurred in 63 (84.0%) of the 75 rivaroxaban recipients at day 22 and in 68 (95.8%) of the 71 rivaroxaban recipients at the end of the intended treatment period. In the 19 control patients, these numbers were 17 (89.5%) and 17 (89.5%), respectively.

In total, 2 (2.7%) patients in the rivaroxaban group had an asymptomatic deterioration at day 22. Both patients continued on rivaroxaban and results normalized at the end of treatment. One (5.3%) control patient experienced an asymptomatic deterioration between day 22 and the end of treatment.

All results for repeat imaging in DVT and PE patients are given in Table 2 for day 22 and in Table 3 for the end of treatment. In patients with DVT, there were no important differences in the results of repeat imaging between the rivaroxaban dose groups.

**Table 2:** **Results of repeat imaging at day 22**

	**Rivaroxaban 10 mg bid/15 mg od**	**Rivaroxaban 15 mg bid/15 mg od**	**Rivaroxaban combined**	**UFH/warfarin**
**DVT patients**	**N = 23**	**N = 24**	**N = 47**	**N = 12**
Improved or normalized, n (%)	18 (78.3)	18 (75.0)	36 (76.6)	10 (83.3)
*Normalized*	*4 (17.4)*	*6 (25.0)*	*10 (21.3)*	*2 (16.7)*
Unchanged, n (%)	4 (17.4)	5 (20.8)	9 (19.1)	2 (16.7)
Deteriorated, n (%)	1 (4.3)	1 (4.2)	2 (4.3)	0
*Asymptomatic*	*1 (4.3)*	*0*	*1 (2.1)*	*0*
Symptomatic*	*0*	*1 (4.2)*	*1 (2.1)*	*0*
**PE patients**	**–**	**N = 28**	**N = 28**	**N = 7**
Improved or normalized, n (%)	**–**	27 (96.4)	27 (96.4)	7 (100)
*Normalized*		*10 (35.7)*	*10 (35.7)*	*1 (14.3)*
Unchanged, n (%)	**–**	0	0	0
Deteriorated, n (%)	**–**	1 (3.6)	1 (3.6)	0
*Asymptomatic*		*1 (3.6)*	*1 (3.6)*	*0*
Symptomatic*		*0*	*0*	*0*
**DVT and PE patients**	**N = 23**	**N = 52**	**N = 75**	**N = 19**
Improved or normalized, n (%)	18 (78.3)	45 (86.5)	63 (84.0)	17 (89.5)
*Normalized*	*4 (17.4)*	*16 (30.8)*	*20 (26.7)*	*3 (15.8)*
Unchanged, n (%)	4 (17.4)	5 (9.6)	9 (12.0)	2 (10.5)
Deteriorated, n (%)	1 (4.3)	2 (3.8)	3 (4.0)	0
*Asymptomatic*	*1 (4.3)*	*1 (1.9)*	*2 (2.7)*	*0*
Symptomatic*	*0*	*1 (1.9)*	*1 (1.3)*	*0*

bid, twice daily, DVT, deep vein thrombosis; od, once daily; PE, pulmonary embolism; UFH, unfractionated heparin; VTE, venous thromboembolism.

*Symptomatic recurrent VTE during first 22 days.

**Table 3:** **Results of repeat imaging at end of intended treatment period**

	**Rivaroxaban 10 mg bid/15 mg od**	**Rivaroxaban 15 mg bid/15 mg od**	**Rivaroxaban combined**	**UFH/warfarin**
**DVT patients**	**N = 20**	**N = 23**	**N = 43**	**N = 12**
Improved or normalized, n (%)	20 (100)	20 (87.0)	40 (93.0)	11 (91.7)
*Normalized*	*10 (50.0)*	*10 (43.5)*	*20 (46.5)*	*4 (33.3)*
Unchanged, n (%)	0	2 (8.7)	2 (4.7)	1 (8.3)
Deteriorated, n (%)	0	1 (4.3)	1 (2.3)	0
*Asymptomatic*	*0*	*0*	*0*	*0*
Symptomatic*	*0*	*1 (4.3)*	*1 (2.3)*	*0*
**PE patients**	**-**	**N = 28**	**N = 28**	**N = 7**
Improved or normalized, n (%)	-	28 (100)	28 (100)	6 (85.7)
*Normalized*		*24 (85.7)*	*24 (85.7)*	*2 (28.6)*
Unchanged, n (%)	-	0	0	0
Deteriorated, n (%)	-	0	0	1 (14.3)
*Asymptomatic*		*0*	*0*	*1 (14.3)*
Symptomatic*		*0*	*0*	*0*
**DVT and PE patients**	**N = 20**	**N = 51**	**N = 71**	**N = 19**
Improved or normalized, n (%)	20 (100)	48 (94.1)	68 (95.8)	17 (89.5)
*Normalized*	*10 (50.0)*	*34 (66.7)*	*44 (62.0)*	*6 (31.6)*
Unchanged, n (%)	0	2 (3.9)	2 (2.9)	1 (5.3)
Deteriorated, n (%)	0	1 (2.0)	1 (1.4)	1 (5.3)
*Asymptomatic*	*0*	*0*	*0*	*1 (5.3)*
Symptomatic*	*0*	*1 (2.0)*	*1 (1.4)*	*0*

bid, twice daily, DVT, deep vein thrombosis; od, once daily; PE, pulmonary embolism; UFH, unfractionated heparin; VTE, venous thromboembolism.

*Symptomatic recurrent VTE during the entire intended treatment period.

## Discussion

The results of this small trial involving Japanese patients with acute symptomatic PE and/or DVT suggest that the doses of rivaroxaban studied yield results similar to that of standard therapy according to Japanese clinical guidelines. The lower limit of the confidence interval (i.e. –3.4%) around the absolute difference in the composite efficacy outcome suggests that an important deterioration in treatment effect can be excluded for rivaroxaban. There were no cases of major bleeding during the assigned study medication periods in either treatment group, and the incidence of other bleeding complications was low and similar in both groups.

Some limitations of our study should be noted. Firstly, we used an open-label design that could have biased the assessment of outcomes. Nevertheless, efforts were made to limit investigator bias, including the requirement to use objective and validated tests to confirm suspected recurrent VTE and the use of an independent committee, whose members were blinded to treatment assignment, to adjudicate outcome events. Secondly, in the control arm the choice of initial parenteral therapy was limited to UFH, which might be regarded as less optimal than LMWH [23]. However, LMWH has not been approved for VTE treatment in Japan. Thirdly, the intensity of vitamin K antagonist treatment used, with a target INR of 2.0 and a range of 1.5–2.5, was lower than that recommended worldwide [4]. In our study, the mean percentage of time during which the INR was in, above, and below the Japanese therapeutic range (1.5–2.5) was 80.3%, 9.3%, and 10.4%, respectively. Although these numbers impress as highly accurate, it should be noted that the corresponding mean percentages of time according to the worldwide recommended INR range of 2.0–3.0 were only 32.5%, 2.7%, and 64.8%, respectively, compared to 61.7%, 16.0% and 22.3%, respectively, in the pooled global EINSTEIN studies [12]. Finally, all 3 patients from a single center were excluded from all analyses because of serious non-compliance to the protocol/Good Clinical Practice guidelines. These patients were assigned to rivaroxaban treatment. Symptomatic recurrent VTE, asymptomatic deterioration, major bleeding, clinically relevant non-major bleeding, or death did not occur in these patients during the study.

There was a slight imbalance in the incidence of thrombophilia between the rivaroxaban- and UFH/warfarin-treated patients. Because thrombophilia is not a risk factor for VTE recurrence during anticoagulant treatment, this difference did not influence the study results. There were no symptomatic recurrent events in the control arm, and the changes in thrombotic burden compare well with those of the global dose-ranging studies with rivaroxaban [17,24]. In the global EINSTEIN dose-finding study [17], a total of 543 patients with acute symptomatic DVT were randomized to receive once-daily rivaroxaban (i.e. 20 mg, 30 mg, or 40 mg) or standard therapy with LMWH and vitamin K antagonist. The primary efficacy outcome was the 3-month incidence of the composite of symptomatic venous thromboembolic complications and asymptomatic deterioration in thrombotic burden, as assessed by comparison of ultrasound and perfusion lung scanning at day 84 with baseline. The primary efficacy outcome occurred in 6.1%, 5.4%, and 6.6% of patients in the rivaroxaban 20, 30, and 40 mg treatment groups, respectively, and in 9.9% of those receiving standard therapy, whereas major bleeding occurred in 0.7%, 1.5%, 0%, and 1.5%, of these rivaroxaban patients, respectively.

## Conclusions

The findings of this study in Japanese patients with acute DVT and/or PE suggest similar efficacy and safety profiles of rivaroxaban and control treatment regimens, consistent with the findings of the worldwide EINSTEIN DVT and PE program.

## Appendix

Members and study centers of the J-EINSTEIN Investigators are listed in the supplementary information (Additional file 1).

## Additional file

Additional file 1:
**List of principal investigators and study centers of the J-EINSTEIN DVT and PE program.**

## Abbreviations

CT: Computed tomography
DVT: Deep-vein thrombosis
INR: International normalized ratio
LMWH: Low molecular weight heparin
PE: Pulmonary embolism
UFH: Unfractionated heparin
VTE: Venous thromboembolism
