# Erratum to: ‘Oral rivaroxaban for Japanese patients with symptomatic venous thromboembolism – the J-EINSTEIN DVT and PE program’

**DOI:** 10.1186/s12959-016-0085-1

**Published:** 2016-05-23

**Authors:** Norikazu Yamada, Atsushi Hirayama, Hideaki Maeda, Satoru Sakagami, Hiroo Shikata, Martin H. Prins, Anthonie W. A. Lensing, Masaharu Kato, Junichi Onuma, Yuki Miyamoto, Kazuma Iekushi, Mariko Kajikawa

**Affiliations:** Department of Cardiology and Nephrology, Mie University Graduate School of Medicine, Mie, Japan; Division of Cardiology, Department of Medicine, Nihon University School of Medicine, Tokyo, Japan; Division of Cardiovascular, Respiratory and general surgery, Nihon University School of Medicine, Tokyo, Japan; Department of Cardiology, National Hospital Organization, Kanazawa Medical Center, Kanazawa, Japan; Department of Cardiovascular Surgery, Kanazawa Medical University, Ishikawa, Japan; Maastricht University Medical Center, Maastricht, The Netherlands; Bayer HealthCare Pharmaceuticals, Wuppertal, Germany; Bayer Yakuhin Ltd, Osaka, Japan

Unfortunately, the original version of this article [1] contained errors. The errors have been addressed point by point in full detail below.

Correction 1

Regarding primary efficacy endpoint (symptomatic recurrent VTE) and composite outcome of symptomatic recurrent VTE or asymptomatic deterioration in rivaroxaban group, we’ve found “1.3 %” (1 patient in 78 population), is correct instead of current “1.4 %”, it should be replaced in Page 1, Line 16 in the abstract section, and on the right side of Page 5, Line 1. However, it is not necessary to replace the number “1.4%” in Table 3 because it was calculated by another definition. Therefore we also would like to add a description of the excluded population, “patients assessed as “not evaluable” were excluded from these analyses”, in footnotes of Table 2 and Table 3.

Correction 2

Due to changes made in correction 1, we need to recalculate absolute risk differences and 95 % Confidence Interval of composite outcome of symptomatic recurrent VTE or asymptomatic deterioration, and we consider these numbers also need to be corrected. And to clarify this “difference” means rivaroxaban reduces risk compared to control, we would like to choose the absolute risk “reduction” instead of “difference”. Absolute risk “reduction” was 4.0 % compared to control, and 95 % CI was (-2.9 to 24.0). We should change these numbers in Page 1, Line 17 in the abstract section, in the right side of Page 5, Line 2 and in the right side of Page 6, Line 6.

Correction 3

There was a trivial miscalculation of percentage in the Table 3. Rivaroxaban combined result in DVT and PE patients, 2 patients in 71 was evaluated as “unchanged”, therefore percentage is “2.8 %” instead of current “2.9 %”.

**Table:** 

	Old text	New text
Abstract Page 1 Line 15 to 17	The composite of symptomatic venous thromboembolic events or asymptomatic deterioration occurred in 1 (1.4 %) rivaroxaban patient and in 1 (5.3 %) UFH/warfarin patient (absolute risk difference, 3.9 % [95 % confidence interval, -3.4–23.8]).	The composite of symptomatic venous thromboembolic events or asymptomatic deterioration occurred in 1 (1.3 %) rivaroxaban patient and in 1 (5.3 %) UFH/warfarin patient (absolute risk reduction, 4.0 % [95 % confidence interval, -2.9-24.0]).
Method Right side Page 3 Line 23 to 25	Crude percentages and absolute differences and their 95 % confidence intervals were calculated.	Crude percentages and absolute reduction and their 95 % confidence intervals were calculated.
Result Left side Page 5 Line 2 to 4	A single patient in the rivaroxaban group (1/78; 1.4 %) developed symptomatic recurrent VTE compared with none of the 19 patients allocated to control treatment.	A single patient in the rivaroxaban group (1/78; 1.3 %) developed symptomatic recurrent VTE compared with none of the 19 patients allocated to control treatment.
Result Left side Page 5 Line 9 to 12	VTE and asymptomatic deterioration at the end of intended treatment occurred in 1 (1.4 %) rivaroxaban patient and in 1 (5.3 %) control patient, with an absolute risk difference of 3.9 % (95 % confidence interval −3.4 to 23.8).	VTE and asymptomatic deterioration at the end of intended treatment occurred in 1 (1.3 %) rivaroxaban patient and in 1 (5.3 %) control patient, with an absolute risk reduction of 4.0 % (95 % confidence interval −2.9 to 24.0) compared to control.
Result Right side Page 5 Line 5 to 8	At day 22, the combined venous ultrasound and lung imaging result in patients with DVT and/or PE showed normalization in 20 (26.7 %) of the 75 rivaroxaban recipients and in 3 (15.8 %) of the 19 control patients.	At day 22, the combined venous ultrasound and lung imaging result in evaluable patients with DVT and/or PE showed normalization in 20 (26.7 %) of the 75 rivaroxaban recipients and in 3 (15.8 %) of the 19 control patients.
Result Page 5 Table 2 Footnote	bid, twice daily, DVT, deep vein thrombosis; od, once daily; PE, pulmonary embolism; UFH, unfractionated heparin; VTE, venous thromboembolism. *Symptomatic recurrent VTE during first 22 days.	bid, twice daily, DVT, deep vein thrombosis; od, once daily; PE, pulmonary embolism; UFH, unfractionated heparin; VTE, venous thromboembolism. Patients assessed as "not evaluable" were excluded from these analyses. *Symptomatic recurrent VTE during first 22 days.
Result Page 6, Table 3 Line 21	2 (2.9)	2 (2.8)
Result Page 6 Table 3 Footnote	bid, twice daily, DVT, deep vein thrombosis; od, once daily; PE, pulmonary embolism; UFH, unfractionated heparin; VTE, venous thromboembolism. *Symptomatic recurrent VTE during the entire intended treatment period.	bid, twice daily, DVT, deep vein thrombosis; od, once daily; PE, pulmonary embolism; UFH, unfractionated heparin; VTE, venous thromboembolism. Patients assessed as "not evaluable" were excluded from these analyses. *Symptomatic recurrent VTE during the entire intended treatment period.
Discussion Page 6 Left side Line 9 to 12	The lower limit of the confidence interval (i.e. –3.4 %) around the absolute difference in the composite efficacy outcome suggests that an important deterioration in treatment effect can be excluded for rivaroxaban.	The lower limit of the confidence interval (i.e. –2.9 %) around the absolute reduction in the composite efficacy outcome suggests that an important deterioration in treatment effect can be excluded for rivaroxaban.
