# Supplementary material for: Oral rivaroxaban for Japanese patients with symptomatic venous thromboembolism – the J-EINSTEIN DVT and PE program
Source: Thromb J. 2015 Jan 17;13:2. doi: 10.1186/s12959-015-0035-3 (PMC4339301; doi:10.1186/s12959-015-0035-3)
Supplement: Additional file 1: — List of principal investigators and study centers of the J-EINSTEIN DVT and PE program. [file 12959_2015_35_MOESM1_ESM.docx]

Supplementary Appendix

**List of principal investigators and study centers**

Principal investigators and study centers participating in the J-EINSTEIN PE study (listed alphabetically by last name): Yoshihisa Aida, Department of Cardiology, Aomori Prefectural Central Hospital; Motomi Ando, Department of Cardiovascular Surgery, Fujita Health University; Kazuhiko Hanzawa, Division of Thoracic and Cardiovascular Surgery, Niigata University Graduate School of Medical and Dental Sciences; Shinji Hasegawa, Department of Cardiology, Japan Community Health care Organization Osaka Hosapital; Masaru Hatano, Department of Cardiovascular Medicine, Graduate School of Medicine, University of Tokyo; Shoji Hirasawa, Cardiovascular medicine, Shizuoka City Shimizu Hospital; Atsushi Hirayama, Division of Cardiology, Department of Medicine, Nihon University School of Medicine; Moriaki Inoko, Cardiovascular Medicine, The Tazuke Kofukai Medical Research Institute, Kitano Hospital; Mitsuaki Isobe, Department of Cardiovascular Medicine, Tokyo Medical and Dental University; Kazunori Iwade, Department of Cardiology, National Hospital Organization Yokohama Medical Center; Kenji Kada, Division of Cardiology, Social Insurance Chukyo Hospital; Nobuyuki Kobayashi, Department of Respiratory medicine, National Center for Global Health and Medicine Hospital; Atsushi Konta, Department of Cardiology, Aomori Prefectural Central Hospital; Yoshihiko Momiyama, Department of Cardiology, National Hospital Organization Tokyo Medical Center; Yasuhiro Morikami, Division of Cardiology, Kumamoto City Hospital; Mikio Mukai, Department of cardiology, Osaka Medical Center for Cancer and Cardiovascular Diseases; Hironori Murakami, Cardiovascular Center Teine Keijinai Hospital; Kozo Naito, Department of Cardiovascular Surgery, Saga Prefectural Medical Center Koseikan; Norifumi Nakanishi, Departments of Cardiology and Radiology, National Cerebral and Cardiovascular Center; Yutaro Nishi, Department of Cardiology, St Luke's International Hospital; Mafumi Owa, Department of Cardiovascular Medicine, Japanese Red Cross Society Suwa Hospital; Satoru Sakagami, Department of Cardiology, National Hospital Organization, Kanazawa Medical Center; Shunpei Sakurai, Department of Cardiology, Aizawa Hospital; Ryotaro Seki, Department of Cardiovascular Medicine, Gunma University School of Medicine; Hiroo Shikata, Department of Cardiovascular Surgery, Kanazawa Medical University, Ishikawa, Japan; Kazuhiro Shimizu, Department of Cardiovascular Center, Sakura Hospital, Toho University Medical Center; Noriaki Takama, Department of Cardiovascular Medicine, Gunma University School of Medicine; Nobuhiro Tanabe, Department of Respirology, Graduate School of Medicine, Chiba University; Hideki Tashiro, Department of Cardiology, St. Mary's Hospital; Hiroshi Tsutsui, Department of Cardiovascular Medicine, Japanese Red Cross Society Suwa Hospital; Shuichi Ueno, Division of Cardiovascular Medicine, Jichi Medical University School of Medicine; Norikazu Yamada, Department of Cardiology and Nephrology, Mie University Graduate School of Medicine; Masao Yamasaki, Department of Cardiology, NTT Medical Center Tokyo; Atsushi Yao, Department of Cardiovascular Medicine, Graduate School of Medicine, University of Tokyo; Fuminobu Yoshimachi, Department of Cardiology, Aomori Prefectural Central Hospital.

Principal investigators and study centers participating in the J-EINSTEIN DVT study (listed alphabetically by last name): Yoshihisa Aida, Department of Cardiology, Aomori Prefectural Central Hospital; Motomi Ando, Department of Cardiovascular Surgery, Fujita Health University; Hitoshi Fujiwara, Department of Cardiology, Hiroshima-Nishi Medical Center; Kazuhiko Hanzawa, Division of Thoracic and Cardiovascular Surgery, Niigata University Graduate School of Medical and Dental Sciences; Shoji Hirasawa, Cardiovascular medicine, Shizuoka City Shimizu Hospital; Moriaki Inoko, Cardiovascular Medicine, The Tazuke Kofukai Medical Research Institute, Kitano Hospital; Hiroyuki Ito, Department of Surgery, Saiseikai Fukuoka General Hospital; Kazunori Iwade, Department of Cardiology, National Hospital Organization Yokohama Medical Center; Masahiro Iwahashi, Department of Cardiovascular surgery, Saiseikai Wakayama Hospital; Masayoshi Kobayashi, Department of Cardiovascular Surgery, Fujita Health University Graduate School of Medicine; Nobuyuki Kobayashi, Department of Respiratory Medicine, National Center for Global Health and Medicine Hospital; Atsushi Konta, Department of Cardiology, Aomori Prefectural Central Hospital; Hideaki Maeda, Division of Cardiovascular, Respiratory and general surgery, Nihon University School of Medicine; Yasushi Matsumoto, Department of Cardiovascular surgery, Kanazawa Medical Center; Yuji Matsumoto, Department of Cardiology, Sasebo City General Hospital; Tetsuro Miyata, Division of Vascular Surgery, Department of Surgery, Graduate School of Medicine, The University of Tokyo; Yoshihiko Momiyama, Department of Cardiology, National Hospital Organization Tokyo Medical Center; Yasuhiro Morikami, Division of Cardiology, Kumamoto City Hospital; Mikio Mukai, Department of cardiology, Osaka Medical Center for Cancer and Cardiovascular Diseases; Mitsuru Munemasa, Division of Cardiology, National Hospital Organization Okayama Medical Center; Hironori Murakami, Cardiovascular Center Teine Keijinai Hospital; Yutaro Nishi, Department of Cardiology, St Luke's International Hospital; Toshiya Okajima, Department of Cardiology, Takarazuka City Hospital; Masataka Sata, Department of Cardiovascular Medicine, Tokushima University Hospital; Ryotaro Seki, Department of Cardiovascular Medicine, Gunma University School of Medicine; Hiroo Shikata, Department of Cardiovascular Surgery, Kanazawa Medical University; Kazuhiro Shimizu, Department of Cardiovascular Center, Sakura Hospital, Toho University Medical Center; Noriaki Takama, Department of Cardiovascular Medicine, Gunma University School of Medicine; Norikazu Yamada, Department of Cardiology and Nephrology, Mie University Graduate School of Medicine; Masao Yamasaki, Department of Cardiology, NTT Medical Center Tokyo; Chikao Yasuda, Department of Surgery, Kinki University School of Medicine; Satoshi Yasuda, Department of Cardiovascular Medicine, National Cerebral and Cardiovascular Center; Fuminobu Yoshimachi, Department of Cardiology, Aomori Prefectural Central Hospital.
